# Forming COVID-19 Policy Under Uncertainty

**DOI:** 10.1017/bca.2020.20

**Published:** 2020-08-06

**Authors:** Charles F. Manski

**Affiliations:** * Department of Economics and Institute for Policy Research, Northwestern University, Evanston, IL, USA, e-mail: cfmanski@northwestern.edu

**Keywords:** coronavirus pandemic, adaptive policy diversification, integrated policy assessment, COVID-19 data uncertainties, D61, D81, I18

## Abstract

This paper presents my thinking and concerns about formation of COVID-19 policy. Policy formation must cope with substantial uncertainties about the nature of the disease, the dynamics of transmission, and behavioral responses. Data uncertainties limit our knowledge of the past trajectory and current state of the pandemic. Data and modeling uncertainties limit our ability to predict the impacts of alternative policies. I explain why current epidemiological and macroeconomic modeling cannot deliver realistically optimal policy. I describe my recent work quantifying basic data uncertainties that make policy analysis difficult. I discuss approaches for policy choice under uncertainty and suggest adaptive policy diversification.

## 1. Introduction

Throughout my career, I have performed econometric research and decision analysis that seeks to characterize and cope with uncertainties that arise in evaluation of public policy. During the past 10 years, I have particularly studied patient care under uncertainty, considering both clinical decision making and population health policy. Manski (2013*a*; 2019*a*) exposits my work on these subjects.

With the onset of the coronavirus pandemic, I am seeking to bring my research to bear. This paper describes my current thinking and concerns. While I focus on COVID-19 policy, much that I write here is relevant to benefit–cost analysis more widely, as uncertainty is a pervasive problem in policy evaluation.

To summarize my main points up front, formation of COVID-19 policy must cope with substantial uncertainties about the nature of the disease, the dynamics of transmission, and behavioral responses. Data uncertainties limit our knowledge of the past trajectory and current state of the pandemic. Data and modeling uncertainties limit our ability to predict the impacts of alternative policies. These uncertainties have been well-recognized qualitatively, but they have not been well-characterized quantitatively. Credible measurement of COVID uncertainties is needed to make useful predictions of policy impacts and reasonable policy decisions.

I have persistently argued for forthright communication of uncertainty in reporting of official statistics and in research that aims to inform policy (Manski, 2011; 2015; 2019*b*). I have criticized the prevalent practice of policy analysis with *incredible certitude.* Exact predictions of policy outcomes are routine. Expressions of uncertainty are rare. Yet predictions often are fragile, resting on unsupported assumptions and limited data. Thus, certitude is not credible.

Epidemiological models of disease dynamics, sometimes combined with models of macroeconomic dynamics, have been used to reach conclusions about optimal COVID-19 policy. However, researchers have done little to appraise the realism of their models, nor to quantify the many uncertainties. Hence, I see little basis to trust the policy prescriptions that have been put forward.

I am concerned that incredible certitude has been prevalent in both epidemiological and economic modeling. I think it misguided to make policy that is optimal in some conjectured scenario but potentially much less than optimal in reality. It is more prudent to approach COVID-19 policy as a problem in decision making under uncertainty.

Facing up to uncertainty, one recognizes that it is not possible to guarantee choice of optimal policies. Nevertheless, one may still make decisions that are reasonable in well-defined respects. The approach most familiar to economists has been maximization of subjective expected welfare. My research has mainly applied the minimax-regret criterion, which chooses a policy that is uniformly nearest to optimal across the feasible states of nature.

I suggest *adaptive diversification* of COVID-19 policy (Manski, 2020*a*). Adaptive policy diversification was proposed and studied in Manski (2009; 2013*a*). Financial diversification is a familiar recommendation for portfolio allocation. Diversification enables an investor facing uncertain asset returns to limit the potential negative consequences of placing “all eggs in one basket.” Analogously, policy is diversified if a planner facing uncertainty randomly assigns treatment units (persons or locations) to different policies. At a point in time, diversification avoids gross errors in policy-making. Over time, it yields new evidence about policy impacts, as in a randomized trial. As evidence accumulates, a planner can revise the fraction of treatment units assigned to each policy in accord with the available knowledge. This idea is adaptive diversification.

In what follows, Section 2 explains why current epidemiological and macroeconomic modeling cannot deliver realistically optimal COVID-19 policy; my discussion draws on Manski (2020*a*, *b*). Section 3 describes my recent work quantifying two basic data uncertainties that make policy analysis difficult (Manski & Molinari, 2020; Manski, 2020*c*). Section 4 discusses approaches for policy choice under uncertainty. This provides background for consideration of adaptive diversification in Section 5.

## 2. Incredible certitude in epidemiological and macroeconomic modeling of the pandemic

Epidemiological modelers have sought to determine COVID-19 policy that would be optimal from a public health perspective if specified models of disease dynamics were accurate and public health were measured in specified ways. Work by the Imperial College COVID-19 Response Team in London and the IHME COVID-19 Health Service Utilization Forecasting Team (2020) at the University of Washington has been particularly influential. I will use an early report by the Imperial College Team to make some general points.

### 2.1 The March 2020 Imperial College report

On 16 March 2020, the Imperial College COVID-19 Response Team made public a report that provided forecasts of the impact of alternative nonpharmaceutical interventions (NPIs) intended to cope with the COVID-19 pandemic in high-income countries, with focus on Great Britain and the USA (Ferguson *et al.,* 2020). The forecasts were made using a modified version of a simulation model previously developed to support pandemic influenza planning. The Response Team distinguished two broad policy alternatives, *mitigation* and *suppression*, which they described as follows (p. 1):

“Two fundamental strategies are possible: (a) mitigation, which focuses on slowing but not necessarily stopping epidemic spread – reducing peak healthcare demand while protecting those most at risk of severe disease from infection, and (b) suppression, which aims to reverse epidemic growth, reducing case numbers to low levels and maintaining that situation indefinitely.”

Drawing implications from their forecasts, they recommended suppression as the preferred policy option. Media coverage indicated that the report immediately affected policy formation in the UK and the USA, influencing both nations to shift sharply from mitigation strategies to suppression.^1^

Should this policy change have occurred? I would confidently say yes if there were reason to think that the Imperial College report provides a credible integrated assessment of the impacts of alternative policies. Unfortunately, the report explicitly did not make an integrated assessment. Moreover, there is reason to question the credibility of the forecasts that it did offer.

Integrated benefit–cost analysis of COVID-19 policy would consider the full impacts on society of alternative policy options. The Imperial College report did not do this. Comparing mitigation and suppression, the Response Team wrote (p. 2):

“We do not consider the ethical or economic implications of either strategy… Instead we focus on feasibility, with a specific focus on what the likely healthcare system impact of the two approaches would be.”

Considering impacts on the healthcare system is obviously important. Nevertheless, it is difficult to understand how the Response Team could justify drawing policy conclusions based only on consideration of the healthcare system.

From the beginning of the pandemic onward, the public has sought to learn the broad impacts of policy on social welfare, which requires joint consideration of healthcare, the economy, education, and other matters. While some have believed that suppression is the best policy from all perspectives, others have argued the contrary. In the USA, potential tension between health and economic objectives quickly become front page news. As early as March 24, a headline in the *New York Times* was^2^ “Trump Considers Reopening Economy, Over Health Experts’ Objections.” As I write this in the summer, criteria for school re-opening in the fall have become controversial.

Why did not the Imperial College Response Team perform an integrated assessment of the broad impacts of COVID-19 policy? The basic answer is that epidemiological modeling has, since its inception a century ago, mainly been performed by quantitative researchers with backgrounds in medicine and public health. Researchers with these backgrounds have found it natural to focus on health concerns, viewing other aspects of social welfare as matters that may be important but are beyond their purview.

Thus, the Response Team mentioned in passing that (p. 2): “Suppression… carries with it enormous social and economic costs which may themselves have significant impact on health and well-being in the short and longer-term.” Yet they made no attempt to quantify social and economic costs. They effectively ignored them when reaching their policy conclusion.

Indeed, the epidemiological model used by the Response Team did not consider how a pandemic may generate behavioral responses within the population. The Response Team acknowledged verbally that behavioral response may be an important determinant of outcomes, stating (p. 1):

“the impact of many of the NPIs detailed here depends critically on how people respond to their introduction, which is highly likely to vary between countries and even communities. Last, it is highly likely that there would be significant spontaneous changes in population behaviour even in the absence of government-mandated interventions.”

This statement acknowledged that the dynamics of epidemics depend on the decisions that individuals make to protect themselves from infection or ignore the danger. Nevertheless, the Response Team did not model behavioral responses. Instead, they invoked assumptions about the fractions of households who would comply with alternative policies, without justifying the assumptions.

I should note that modeling and analysis of behavioral responses to epidemics has been a central concern of a separate literature on economic epidemiology, whose contributors are primarily health economists rather than researchers with backgrounds in medicine and public health. See Philipson (2000).

### 2.2 Integrated epidemiological and macroeconomic modeling

Following the onset of the pandemic, macroeconomists have sought to expand the scope of optimal policy analysis by joining epidemiological models with models of macroeconomic dynamics and by specifying welfare functions that consider both public health and economic outcomes. See, for example, Acemoglu *et al.* (2020), Eichenbaum *et al.* (2020), and Thunström *et al.* (2020). Research of this type is potentially welcome, but there is little basis to assess the realism of the models that have been developed.

A serious problem in both epidemiological and macroeconomic modeling is the dearth of evidence available to inform model specification and estimation. Studies of infectious disease and macroeconomic dynamics are largely unable to perform the randomized trials that have been considered the so-called “gold standard” for medical research. Modeling necessarily relies on observational data, which can be difficult to interpret even when they are accurate. Moreover, existing data on the COVID-19 pandemic are notoriously inaccurate. Lacking much evidence, epidemiologists and macroeconomists have developed models that may be mathematically sophisticated but that have little grounding.

These modeling efforts may perhaps be useful if interpreted cautiously as computational experiments studying policy making in hypothetical worlds. However, their relevance to the real world is unclear. Models differ considerably in the assumptions they maintain and in the way they use limited available data to estimate parameters. Researchers provide little information that would enable one to assess model realism. They do little to quantify uncertainty in the predictions they offer. Thus, incredible certitude has been prevalent in both epidemiological and economic modeling of the pandemic.

I see an urgent need for epidemiologists and economists to join forces to develop credible integrated assessment models of epidemics. Even with the best intentions, this will take considerable time. There is some reason to hope that epidemiologists and macroeconomists may be able to communicate with one another because they share a common language for mathematical modeling of dynamic processes, used to formalize SIR models and DSGE models respectively. However, each group has in the past exhibited considerable insularity, which may impede collaboration. Moreover, neither discipline has shown much willingness to face up to uncertainty when developing and applying models.

Looking ahead towards credible integrated assessment of COVID-19 policy and public health policy more generally, I see lessons to be learned from research on climate policy. Climate research was at first a subject for study by earth scientists, who seek to forecast the impact of emissions on the atmosphere and oceans. Having backgrounds in the physical sciences, these researchers find it natural to focus on the physics of climate change rather than behavioral responses and social impacts. Over the past 30 years, the study of climate policy has broadened with the development of integrated assessment models, with major contributions by economists (Nordhaus, 2013).

As a result, we now have a reasonably sophisticated perspective on how our physical planet and our social systems interact with one another. This progress has so far been more qualitative than quantitative. Existing integrated assessment models make quantitative forecasts, but the credibility of climate models is still limited (Pindyck, 2017). Climate researchers and covid researchers alike should work to improve the credibility of their modeling.

## 3. Data uncertainties

It is widely appreciated that severe data uncertainties limit our knowledge of the past trajectory and current state of the pandemic. Nevertheless, public health agencies report point estimates of basic statistics such as infection rates and infection-fatality rates. These estimates are commonly taken at face value by policy makers and the public, but they may be highly inaccurate. The result is incredible certitude.

In two recent papers summarized below (Manski & Molinari, 2020; Manski, 2020*c*), I use *partial identification* analysis to obtain credible bounds on basic covid statistics. Study of partial identification removes the traditional focus of econometrics and statistics on point estimation obtained under strong assumptions. It poses weaker assumptions that should be credible in the context under study. Weak assumptions commonly yield bounds rather than point estimates. Strengthening the assumptions narrows the bounds. The methodological problem is to determine the bound that logically results when available data are combined with specified assumptions. See Manski (1995; 2003; 2007) for monograph expositions at different technical levels. See Tamer (2010) and Molinari (2020) for review articles.

### 3.1 Bounding the COVID-19 infection rate

Manski and Molinari (2020) address the serious problem that accurate characterization of the time path of the coronavirus pandemic has been hampered by missing data. Confirmed cases have been measured by rates of positive findings among persons who have been tested for infection. Infection data are missing for persons who have not been tested.

The persons who have been tested differ considerably from those who have not been tested. Criteria used to determine eligibility for testing have typically required demonstration of symptoms associated with presence of infection or close contact with infected persons. This gives considerable reason to believe that some fraction of untested persons is an asymptomatic or presymptomatic carrier of the COVID-19 disease. Hence, the actual cumulative rate of infection has been higher than the reported rate.

A second problem of data quality is that measurement of confirmed cases is imperfect because the prevalent nasal swab tests for infection are not fully accurate. There is basis to think that accuracy of nasal swab tests is highly asymmetric, with few false positive results but many false negative ones. Given this asymmetry, the actual rate of infection has again been higher than the reported rate.

Combining the problems of missing data and imperfect test accuracy yields the conclusion that reported cumulative rates of infections are lower than actual rates. Reported rates of infection have been used as the denominator for computation of rates of severe disease conditional on infection, measured by rates of hospitalization and death. Presuming that the numerators in rates of severe illness conditional on infection have been measured accurately, reported rates of severe illness conditional on infection are higher than actual rates.

Researchers have put forward point estimates for infection rates and rates of severe illness derived in various ways. The estimates differ in the assumptions used. The assumptions vary and so do the findings. No assumption or estimate has been thought sufficiently credible as to achieve consensus.

I think it more informative to determine the range of infection rates and rates of severe illness implied by a credible spectrum of assumptions. Manski and Molinari (2020) combine available data with credible assumptions to bound the cumulative infection rate at specific locations and dates. Knowledge of this statistic is essential to forecast the level of herd immunity that a population has achieved by a certain date. It is also necessary to calculate probabilities of severe illness conditional on infection, including risks of hospitalization and death. Knowledge of these probabilities is vital to inform both personal risk assessment and public health policy.

We explain the logic of the identification problem and we determine the identifying power of some credible assumptions. In particular, we assume that the infection rate among untested persons is lower than the rate among tested persons. We assume a bound on the accuracy of nasal swab tests. Using these and other assumptions, we derive a bound on the population infection rate.

To illustrate, we analyze data from Illinois, New York, and Italy in March and April 2020. We obtain bounds that are wide but yield some information. For example, we find that the cumulative infection rates on April 24 are in the intervals [0.004, 0.525], [0.017, 0.618], and [0.006, 0.471] respectively. The cumulative infection-fatality rates are in the intervals [0, 0.033], [0.001, 0.049], and [0.001, 0.077].

### 3.2 Bounding the accuracy of diagnostic tests, with application to COVID-19 antibody tests

I mentioned above that swab tests for COVID-19 have imperfect accuracy. A *false positive* occurs when a result indicates illness, but the person has not been ill. A *false negative* occurs when a result indicates no illness, but the person has been ill. In general, a medical diagnostic test may be informative about current or past illness. COVID-19 swab tests do the former and antibody tests do the latter. I write “has been ill” to encompass both types of test.

For personal risk assessment, clinical decision making, and measurement of population infection rates, one would like to know the positive and negative predictive values of a test. *Positive predictive value* (PPV) is the chance that a member of a population who tests positive has been ill. Negative predictive value (NPV) is the chance that someone who tests negative has not been ill. Accurate measurement of PPV and NPV is often difficult. Manski (2020*c*) explains why and shows how to derive credible bounds.

Studies of test accuracy regularly report statistics other than PPV and NPV, *sensitivity* and *specificity.* Sensitivity is the chance that an ill person receives a positive test result. Specificity is the chance that a non-ill person receives a negative result. Knowing sensitivity and specificity permits prediction of a test result given true illness status. These predictions are not directly relevant to risk assessment, clinical decisions, or measurement of infection rates. For these purposes, one knows a test result and wants to predict whether a person has been ill. One does not know whether a person has been ill and want to predict a result.

Given that PPV–NPV are socially relevant concepts while sensitivity and specificity are not, it is natural to ask why measurement of test accuracy often focuses on the latter quantities rather than the former. Part of the answer appears to be that researchers find it easier to measure sensitivity and specificity. Estimation of PPV–NPV requires observation of test results and true illness status for a representative sample of the relevant population, say through random sampling. It may be easy to observe test results but not true illness status. If it were easy to observe true illness status, tests would serve no practical purpose.

Whereas observation of true illness status may be difficult in general, researchers sometimes do so for special groups of persons. This enables estimation of sensitivity and specificity for these groups. The practice has been to estimate in this manner and assume that the findings obtained for the special groups hold in the relevant population.

Suppose that sensitivity and specificity have been estimated in some groups and that one finds it credible to extrapolate to the relevant population. Then PPV–NPV can be derived via Bayes Theorem if one knows the *prevalence* of the disease; that is, the rate of illness in the population. Unfortunately, it is often difficult to measure prevalence.

COVID-19 presents a case of concern. There are now two main classes of tests for COVID-19. Swab tests detect the presence of live virus, signaling an active infection. Serological tests detect the presence of antibodies that the immune system develops after onset of infection. The presence of antibodies signals that a person was infected in the past. Prevalence is the current population infection rate when testing for active infection and is the cumulative infection rate when testing to detect antibodies.

It is appreciated that these infection rates are highly uncertain, for the reasons discussed in Section 3.1. To derive estimates of PPV–NPV for antibody tests, the U.S. Food and Drug Administration (FDA) assumed that the cumulative infection rate is 0.05 (U.S. Food and Drug Administration, 2020). However, the FDA recognized that this assumption lacks foundation, stating: “We do not currently know the prevalence of severe acute respiratory syndrome coronavirus 2 (SARS-CoV-2) antibody positive individuals in the U.S. population, and prevalence may change based on the duration the virus is in the country and the effectiveness of mitigations.”

To cope with difficulty in measurement of prevalence, epidemiologists have developed methods that yield frequentist point estimates or Bayesian posterior distributions under alternative assumptions. However, the uncertainties in settings such as COVID-19 make it difficult to justify assumptions generating point estimates or posterior distributions. Rather than make such assumptions, Manski (2020*c*) studies partial identification of PPV–NPV given credible bounds on prevalence, such as those obtained in Manski and Molinari (2020). Applying the methodology to COVID-19 antibody tests authorized by the FDA, I obtain narrow bounds for NPV and wide bounds for PPV with the current limited knowledge of prevalence.

## 4. Criteria for reasonable policy choices under uncertainty

Juxtaposing the modeling and data uncertainties described in Sections 2 and 3, I think it essential for benefit–cost analysis to view formation of COVID-19 policy as a problem of decision making under uncertainty. Modeling and data uncertainties express incomplete knowledge of the real world, what decision theorists call the *state of nature.* Benefit–cost analysis also has to cope with normative uncertainties about the social welfare function that should be used to measure benefits and costs. Perennially controversial questions including the appropriate rate of time discount and assessment of value-of-life. Dudley *et al.* (2019) provide perspectives on multiple sources of uncertainty. In this paper, I suppose that the social welfare function has been chosen and I focus on uncertainty about the state of nature.

The standard formalization of decision making under uncertainty supposes that a decision maker chooses among a set of feasible actions. The welfare achieved by any action depends on the state of nature. The decision maker lists all states that he believes could possibly occur. This list, the *state space*, expresses partial knowledge. The larger the state space, the less the decision maker knows about the outcome of each action.

The fundamental difficulty of decision making under uncertainty is clear even in a simple setting with two feasible actions and two states of nature. Suppose that one action yields higher welfare in one state of nature and the other action yields higher welfare in the other state. Then the decision maker does not know which action is better. Thus, optimization is impossible. Ferguson (1967) put it this way (p. 28):

“It is a natural reaction to search for a ‘best’ decision rule, a rule that has the smallest risk no matter what the true state of nature. Unfortunately, *situations in which a best decision rule exists are rare and uninteresting.* For each fixed state of nature there may be a best action for the statistician to take. However, this best action will differ, in general, for different states of nature, so that no one action can be presumed best overall.”

Decision theory suggests a two-step decision process. One first eliminates dominated treatments: an action is dominated if one knows that some other one is at least as good in all feasible states of nature and superior in some state. One then chooses an undominated action. This is subtle because there is no optimal way to choose among undominated alternatives. There are only various reasonable ways, each with its own properties. The word “reasonable” is not easy to pin down. Ferguson (1967) wrote (p. 29): “A *reasonable* rule is one that is better than just guessing.”

### 4.1 Maximization of subjective expected welfare

What are specific reasonable ways to make an undominated choice? Most familiar to economists is placement of a subjective probability distribution on the state space and maximization of subjective expected welfare. These are often called Bayes decisions. This approach was taken by Nordhaus (2013) and elsewhere in his integrated assessments of climate policy. It could similarly be used to perform integrated assessment of COVID-19 policy.

Bayesian decision making is compelling when one feels able to place a credible subjective distribution on the state space. However, a subjective distribution is a form of knowledge, and a decision maker may not feel able to assert one. Bayesians have long struggled to provide guidance and the matter continues to be controversial. The controversy suggests that inability to express a credible subjective distribution is common in actual decision settings.

When one finds it difficult to assert a credible subjective distribution, Bayesians may suggest use of some default distribution, called a “reference” or “conventional” or “objective” prior. However, there is no consensus on the prior that should play this role. The chosen prior affects decisions.

### 4.2 Criteria achieving uniformly satisfactory decisions

When one finds it difficult to assert a credible subjective distribution, a reasonable way to act is to use a decision criterion that achieves uniformly satisfactory results, whatever the true state of nature may be. There are two prominent ways to formalize the idea of uniformly satisfactory results, maximin and minimax-regret (MMR) decision making.

The maximin criterion chooses an action that maximizes the minimum welfare that might possibly occur. The minimax-regret criterion considers each state of nature and computes the loss in welfare that would occur if one were to choose a specified action rather than the one that is best in this state. This quantity, called *regret*, measures the nearness to optimality of the specified action in the state of nature. The decision maker must choose without knowing the true state. To achieve a uniformly satisfactory result, he computes the maximum regret of each action; that is, the maximum distance from optimality that the action would yield across all possible states of nature. The MMR criterion chooses an action that minimizes this maximum distance from optimality.

The maximin and MMR criteria are sometimes confused with one another, but they yield the same choice only in certain special cases. The former chooses an action that maximizes the minimum welfare that might possibly occur. The latter chooses an action that minimizes the maximum loss to welfare that can possibly result from not knowing the welfare function. Thus, whereas the maximin criterion considers only the worst outcome that an action may yield, MMR considers the worst outcome relative to what is achievable in a given state of nature.

I have applied the maximin and MMR criteria to study many problems of policy formation, emphasizing MMR. The contexts have included policing (Manski, 2006; Manski & Nagin, 2017), bank regulation (Brock & Manski, 2011), income taxation (Manski, 2014), vaccination (Manski, 2010; 2017), and clinical treatment choice (Manski, 2009; 2013*b*; 2018; Cassidy & Manski, 2019; Manski & Tetenov, 2020). These criteria should similarly be applicable to formation of COVID-19 policy.^3^

## 5. Adaptive diversification of COVID-19 policy

As I write this during the continuing pandemic, I am unaware of benefit–cost analysis that uses the decision theory described above to inform choice of COVID-19 policy under uncertainty. This is unfortunate because the need is urgent. I can, however, suggest application of a broad idea that can be justified both by Bayesian and MMR decision making. This is adaptive policy diversification.

There have been frequent calls for adoption of a uniform COVID-19 policy across locations, particularly across the 50 states of the USA. For example, a 11 May 2020 editorial in the *Washington Post* ^4^ was titled “The patchwork of state reopenings is a deadly game of trial and error.” The text refers to “the peril posed by the hodgepodge of state decisions to reopen quickly, gradually or not at all yet.” While warning against decentralization of policy making across the states, the editorial did not propose what a uniform national policy should be.

Calling for a uniform COVID-19 policy across states would be justified if it were clear what constitutes optimal policy and if it were known that the optimal policy is invariant across states. Then each state should adhere to that policy. However, as explained above, we do not know what optimal policy is for any state. It may be that continued suppression is better for some states (or regions) and that some version of reopening is better for others, depending on their characteristics. Hence, I see no prima facie case for making policy uniform across states.

It has long been appreciated in the USA that uncertainty may justify decentralization of policy making, enabling the states to experiment with policy ideas. Supreme Court Justice Louis Brandeis, in his dissent to the 1932 case *New York State Ice Co. v. Liebmann* (285 U.S. 311) made what has become a famous remark on this theme: “It is one of the happy incidents of the federal system that a single courageous State may, if its citizens choose, serve as a laboratory; and try novel social and economic experiments without risk to the rest of the country.” It has since become common to refer to the states as the *laboratories of democracy.*

The Brandeis statement expresses the “adaptive” aspect of the theme of adaptive diversification, recognizing that policy variation across states stimulates learning about policy impacts. The diversification aspect of the theme has been less well appreciated.

To illustrate, consider the choice between suppression and mitigation framed by Ferguson *et al.* (2020). Suppression may be the better policy if the Imperial College model makes reasonably accurate predictions of covid health impacts and if the economic impacts ignored by the model are relatively small. On the other hand, mitigation may be the better policy if the model substantially overestimates the covid health impacts or if the economic impacts ignored by the model are relatively large. Policy diversification, with some locations implementing suppression and others implementing mitigation, gives up the ideal of optimality in order to protect against making a gross error in policy choice. To help inform policy diversification, it would be useful to develop models that enable credible integrated assessment of covid policy at the state or regional level.

When diversifying, what fraction of locations should implement each policy option under consideration? This depends on the welfare function that society uses to evaluate options and on the uncertainties that afflict prediction of policy impacts. Manski (2009) studied adaptive diversification when social welfare is utilitarian, and a planner uses a simple dynamic version of the minimax-regret criterion to cope with uncertainty. The result is a simple diversification rule. Given specification of an appropriate welfare function and characterization of the relevant uncertainties, it should be possible to adapt this analysis to diversify at least some aspects of COVID-19 policy.

A caveat regarding implementation of adaptive diversification in the USA is that variation of COVID-19 policy across states is not the result of purposeful randomization. It is the result of state-specific decision processes. Federalism empowers the states to choose their own public health policies.

Nevertheless, federalism does not require that the federal government remain passive. The federal government can provide incentives to the states to encourage them to enact desirable portfolios of policies. Thus, the federal government can encourage adaptive diversification across states, modifying the incentives as knowledge accumulates. The federal government played such an active role in welfare policy in the late 1980s, when it encouraged states to institute and evaluate variations on the then-existing program of Aid for Families with Dependent Children (Manski & Garfinkel, 1992).

## Footnotes

1 See, for example, https://www.nytimes.com/2020/03/17/world/europe/coronavirus-imperial-college-johnson.html.

2
https://www.nytimes.com/2020/03/23/business/trump-coronavirus-economy.html?action=click&module=Spotlight&pgtype=Homepage

3 One might also apply hybrid criteria that use a partial subjective distribution on the states of nature. A planner who asserts a partial subjective distribution could maximize minimum subjective average welfare or minimize maximum average regret. These criteria combine elements of averaging across states and concern with uniform performance across states. Statistical decision theorists refer to these criteria as Γ-maximin and Γ-minimax regret (Berger, 1985). The former criterion has drawn attention from axiomatic decision theorists, with the terminology max–min expected utility (Gilboa & Schmeidler, 1989).

4
https://www.washingtonpost.com/opinions/the-patchwork-of-state-reopenings-is-a-deadly-game-of-trial-and-error/2020/05/11/5e255288-9179-11ea-a0bc-4e9ad4866d21_story.html.
